# Chemical Composition, Antitumor Properties, and Mechanism of the Essential Oil from *Plagiomnium acutum* T. Kop.

**DOI:** 10.3390/ijms232314790

**Published:** 2022-11-26

**Authors:** Meiya Li, Linyan Wang, Shiqing Li, Chenglong Hua, Hang Gao, Dandan Ning, Changyu Li, Chunchun Zhang, Fusheng Jiang

**Affiliations:** 1 Academy of Chinese Medical Sciences, Zhejiang Chinese Medical University, Hangzhou 310053, China; 2 College of Pharmaceutical Science, Zhejiang Chinese Medical University, Hangzhou 310053, China; 3 College of Life Science, Zhejiang Chinese Medical University, Hangzhou 310053, China

**Keywords:** *Plagiomnium acutum* T. Kop., essential oil, antitumor activity, apoptosis, terpenoids, mitochondria

## Abstract

*Plagiomnium acutum* T. Kop. (*P. acutum*) has been used as a traditional Chinese medicine for thousands of years to treat cancer but lacks evidence. The objective of this work was to reveal the chemical composition of *P. acutum* essential oil (PEO) and explore its potential antitumor activity and molecular mechanism. PEO was prepared by the simultaneous distillation–extraction method and characterized by gas chromatography/mass spectroscopy. CCK8 assay, flow cytometry, western blot, and immunofluorescence techniques were used to analyze the effects and mechanism of PEO against cancer cells. A total of 74 constituents of PEO were identified, with diterpenes (26.5%), sesquiterpenes (23.89%), and alcohols (21.81%) being the major constituents. Two terpenoids, selina-6-en-4-ol and dolabella-3,7-dien-18-ol, were detected in PEO for the first time. PEO showed significant cell growth inhibitory activity on HepG2 and A549 cells by blocking the G1 phase and inducing apoptosis, which may be attributed to its upregulation of p21^Cip1^ and p27^Kip1^ proteins and interference with mitochondrial membrane potential effect. Dolabella-3,7-dien-18-ol accounts for 25.5% of PEO and is one of the main active components of PEO, with IC_50_ values in HepG2 and A549 cells of (25.820 ± 0.216) µg/mL and (23.597 ± 1.207) μg/mL, respectively. These results confirmed the antitumor medicinal value of *P. acutum* and showed great application potential in the pharmaceutical industry.

## 1. Introduction

Essential oils are valuable volatile substances naturally produced by plants. They are produced almost by all parts of plants as secondary metabolites and are well known for their antiseptic (bactericidal, fungicidal, and virucidal), insecticidal, medicinal, and aromatic properties [1]. To date, approximately 3000 essential oils have been identified, more than 300 of which have high commercial value, particularly in the food, sanitary, cosmetics, perfume, and pharmaceutical industries [2]. Numerous research reports on the composition, efficacy, and application of essential oils are published each year. These studies improve the value of plant crops, bring economic benefits to farmers, and promote human health.

Bryophytes represent the second largest plant group after angiosperms and can be found almost everywhere in the world except in the sea. Worldwide, there are approximately 23,000 species of bryophytes, which can be divided into three phyla, mosses (Bryophyta), liverworts (Marchantiophyta), and hornworts (Anthocerotophyta) [3]. Bryophytes have a wide range of medicinal value. In particular, mosses have been used as medicinal plants in China for thousands of years for the treatment of trauma, fractures, burns, snake bites, hemorrhage, neurasthenia, pneumonia, tuberculosis, convulsions, cancer, and urinary system diseases [4,5,6]. North American Indians used some moss species for the treatment of burns, bruises, and wounds [7]. Phytochemistry studies have shown that mosses produce a variety of volatile metabolites, particularly terpenoids and aromatic compounds [3,8,9,10]. The chemical constituents and biological activities of liverworts, one of the oldest terrestrial bryophytes, have been more extensively studied than those of mosses. To date, over 1600 terpenoids have been isolated and identified from liverworts, and over 40 new carbon skeletons of terpenoids and aromatic compounds have been found [7,8]. These compounds exhibit remarkable biological activities including antibacterial, antifungal, antiviral, anti-inflammatory, antioxidant, cytotoxic, muscle relaxing, neurotrophic, insect repellant, and plant growth-regulatory activities, and some compounds have potential cosmetic and pharmaceutical uses [7,8].

The moss *Plagiomnium acutum* T. Kop. (*P. acutum*) belongs to the Mniaceae family and is an important indicator of environmental pollution [11] along with the overwintering host of gallnut aphids [9]. *P. acutum* is easy to cultivate [12] and has great economic value in landscaping [12,13]. Additionally, *P. acutum* has been used to treat epistaxis, metrorrhagia, and cancer for thousands of years in Chinese traditional and folk medicine [6]. The essential oil of *P. acutum* is abundant in aliphatic derivatives and terpenoids [9,10]. However, its pharmacological activities have been scarcely studied. In this work, we identified a total of 74 constituents in the *P. acutum* essential oil by gas chromatography/mass spectroscopy (GC/MS). Two of the identified terpenoids, selina-6-en-4-ol and dolabella-3,7-dien-18-ol, were reported in the essential oil of moss for the first time. In particular, the compound dolabella-3,7-dien-18-ol accounted for 25.5% of the total essential oil and showed significant antitumor activity. Therefore, the current work investigated the potential antitumor activity of *P. acutum* essential oil along with the related mechanism.

## 2. Results and Discussion

### 2.1. Composition of PEO

**Figure 1:**
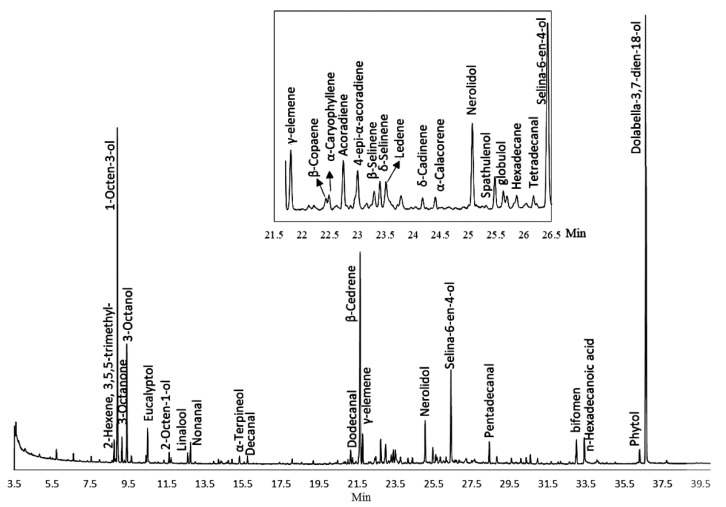
Gas chromatogram mass spectrometry of *P*. *acutum* oil.

The fresh aerial part of *P. acutum* was collected and extracted by simultaneous distillation–extraction (SDE), producing a light-yellow, fragrant essential oil with a yield of (0.0500 ± 0.0018%) (W/fresh weight). The chemical constituents of PEO were analyzed by GC/MS (Table 1 and Figure 1). In total, 74 constituents were identified based on their RI and mass spectra, representing 83.5% of the total PEO compositions. The qualitative and quantitative determination of PEO constituents indicated that diterpenes (26.5%), sesquiterpenes (23.89%), and alcohols (21.81%) were the major constituents. The volatiles of compounds in PEO were abundant in aliphatic alcohols (e.g.,1-octen-3-ol, n-octanol, etc.) and aliphatic aldehydes (e.g., n-heptanal, n-nonanal, pentadecanal, etc.). In addition, various terpenoid compounds were detected in a total proportion of 53.58%, and most of them were readily identified by their characteristic mass spectra and RI. These compounds, which include α-pinene, camphene, β-elemene, α-copaene, linalool, and β-selinene, seem to be almost ubiquitous in mosses. These findings are consistent with previous reports [10,14]. Surprisingly, we detected abundant and highly variable sesquiterpene hydrocarbons; among them, the most abundant compounds were tricyclic sesquiterpenes (52.9%), followed by oxygenated bicyclic sesquiterpenes (18.4%), bicyclic sesquiterpenes (11.7%), monocyclic sesquiterpenes (7.0%), oxygenated acyclic sesquiterpenes (6.1%), and a small amount of oxygenated tricyclic sesquiterpenes (3.8%). The common sesquiterpenes in PEO were β-cedrene (11.39%), acoradiene (0.88%), 4-epi-α-acoradiene (0.94%), germacrene D, (+)-ledene and δ-cadinene, β-nerolidol (1.46%), α-cadinol, and globulol (Table 1). The abundant sesquiterpene alcohol selina-6-en-4-ol (3.82%) was reported as a constituent of the essential oil of *Psidium guajava* Var. Pomifera (Myrtaceae) [15] and *Cinnamomum caphora* [16] but has not been previously reported as a component of moss essential oils.

The compound with an RI of 2133 was 91% similar to cembrenol (RI = 2162) in the NIST 14 library. However, due to their different RI values, this compound was isolated and purified by silica gel column chromatography and identified as dolabella-3,7-dien-18-ol (see Figure S1) by comparing the NMR data of the purified compound with the literature data [18,19]. The spectrometric data for compound dolabella-3,8(17)-dien-18-ol (C_20_H_34_O) were as follows: EI-MS, *m*/*z* 272 (M-H_2_O, 2), 257 (M-H_2_O-CH_3_, 2), 229 (M-H_2_O-3CH_3_, 5), 189 (9), 161 (12), 147 (13), 135 (39), 121 (41), 107 (45), 95 (56), 81 (66), 67 (56), 59 (100). ^1^ H NMR (600 MHz, CDCl_3_), δ 5.00 (1H, t, vinyl), δ 4.89 (1H, t, vinyl), δ 2.07–2.21 (7H, m, methyl), δ1.72 (1H, m, methyl), δ 1.67 (2H, m, methyl), 1.55 (6H, s, methyl), δ 1.51 (2H, m, methyl), δ 1.25 (3H, s, methyl), δ 1.31–1.48 (4H, m, methyl), δ 1.23 (3H, s, methyl), δ 1.0 (3H, s, methyl). ^13^ C NMR (600 MHz, CDCl_3_), δ 16.39, 16.51, 23.19, 24.90, 26.43, 26.50, 30.81, 31.49, 38.72, 39.24, 39.55, 41.09, 41.71, 46.67, 60.23, 73.23, 124.65, 126.53, 132.99, 135.40 ppm.

Surprisingly, dolabella-3,7-dien-18-ol, a dolabellane-type diterpene, is the main component in PEO, accounting for approximately 25.5% of the total essential oil. This is the first report of this compound in the essential oils of *P. acutum* and other mosses [9,10]. In contrast, several dolabellane-type diterpenes have been reported in liverworts [8]. The differences in the volatile components of PEO in comparison to the essential oils of other mosses may result from differences in harvest seasons, geographical locations, subspecies, and processing methods [10]. Numerous dolabellane-type diterpenes show strong antitumor activities [20,21]. Considering the strong antitumor activities of terpenoids and the abundance of terpenoids in PEO, the antitumor activity of PEO was further explored.

### 2.2. In Vitro Cell Cytotoxicity

**Figure 2:**
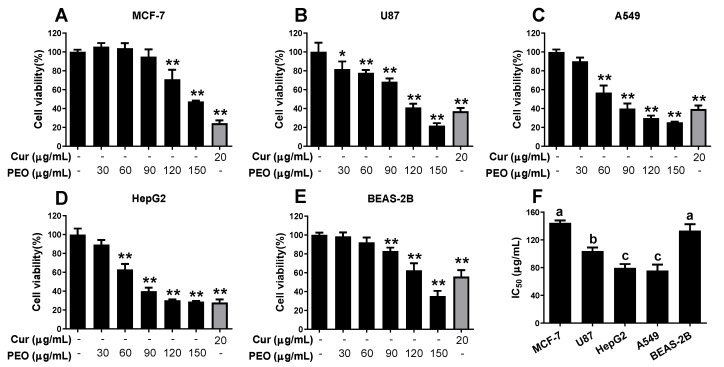
Cytotoxicity of PEO against different tumor cell lines. Four tumor cell lines (**A**) MCF-7, (**B**) U87, (**C**) A549, (**D**) HepG2, and (**E**) one normal human bronchial epithelial cell line BEAS-2B were treated with or without different concentrations of PEO for 24 h, then cell viabilities were determined by CCK8 method. (**F**) The IC_50_ of PEO against five cell lines were calculated. The values represent mean ± standard deviation of three independent experiments. * *p* < 0.05, ** *p* < 0.01 compared with the untreated group. Different letters indicate significant differences, *p* < 0.05.

The cytotoxicity of PEO was evaluated in different cancer cell lines (MCF-7, U87, HepG2, and A549) by cell counting kit-8 (CCK8) assay. PEO inhibited the growth of the four tested cancer cell lines in a dose-dependent manner (Figure 2). The cell lines showed different sensitivities to PEO; the IC_50_ values of A549 and HepG2 cells (75.030 ± 6.947 μg/mL and 79.557 ± 5.533 μg/mL, respectively) were significantly lower than those of U87 and MCF-7 cells (IC_50_ = 104.040 ± 4.990 and 144.667 ± 3.544 μg/mL, respectively). PEO also showed inhibitory activity against the normal human bronchial epithelial cell line BEAS-2B. Compared with A549, HepG2, and U87 cells, the cytotoxicity of PEO against BEAS-2B cells (IC_50_ = 133.533 ± 9.254 μg/mL) was significantly reduced. In contrast, no significant difference was observed between the IC_50_ values of PEO against BEAS-2B and MCF-7 cells. The data indicated that MCF-7 cells had a high tolerance to PEO while A549 and HepG2 cells were more sensitive. Therefore, A549 and HepG2 cells were used in the following experiments. Compared with A549 and HepG2 cells, PEO had lower toxicity in normal human bronchial epithelial cells, indicating its potential antitumor activity.

### 2.3. Real-Time Cell Analysis (RTCA)

**Figure 3:**
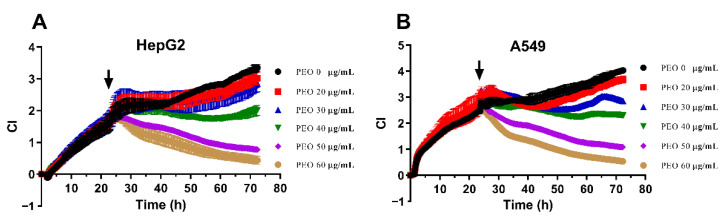
CI curves of (**A**) HepG2 cells and (**B**) A549 cells treated by increasing concentration of PEO. HepG2 and A549 cells were seeded into E-Plate96. E-Plate96 was locked in RTCA-DP device at 37 °C with 5% CO_2_. Twenty hours postplating, cells were treated with different concentrations of PEO. CI was read automatically every 15 min and the recorded curves were normalized to the time point of cell seeding, referred to as normalized CI. All experiments were performed in triplicate. Point of PEO addition is shown by a black arrow.

In addition to inhibiting the growth of tumor cells, PEO also significantly affected the morphology and movement of tumor cells. RTCA provides information about the number of cells along with quantitative data about cell biological status (e.g., cell viability, morphology, and movement) [22]. Therefore, the effects of PEO on A549 and HepG2 cells were further evaluated by RTCA assay. The CI curves for A549 and HepG2 cells exposed to different PEO concentrations are presented in Figure 3. Consistent with the CCK8 results, PEO inhibited the proliferation of A549 and HepG2 cells in a dose- and time-dependent manner, and the IC_50_ values calculated based on the CI values were much smaller than those calculated based on CCK8 assay. It should be noted that the CI values of HepG2 and A549 cells did not increase after treatment with PEO at 40 μg/mL, while the CI values continuously decreased after the addition of PEO at 50 μg/mL or 60 μg/mL. This suggests that PEO treatment at doses of 50 μg/mL or greater inhibited cell proliferation and significantly affected cell morphology and adhesion, which may be related to the PEO-induced shrinkage and apoptosis of cells.

### 2.4. Induction of Cell Apoptosis

The continuous decline in the CI value indicated by RTCA indirectly reflects a change in cell morphology and decrease in cell adhesion, which can be verified by microscope. After PEO treatment, numerous cells became round, shrank, and developed blebs. These morphological changes are closely related to apoptosis [23]. Therefore, we analyzed apoptosis analysis to clarify the mechanism by which PEO inhibits the growth of A549 and HepG2 cells.

#### 2.4.1. PEO-Induced Apoptosis in HepG2 and A549 Cells

**Figure 4:**
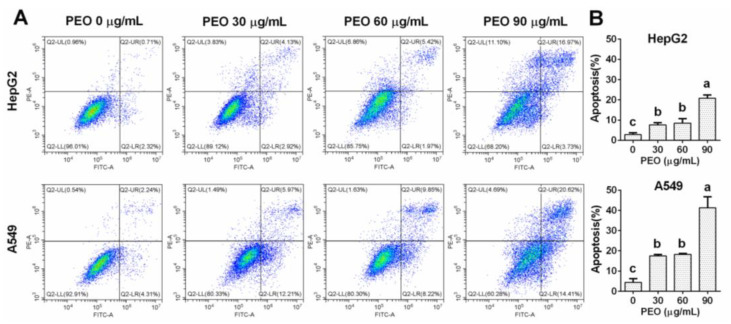
Inducing apoptosis effect of PEO in HepG2 and A549 cells. HepG2 and A549 cells were treated with or without different concentrations of PEO (30, 60, and 90 μg/mL) for 24 h, then cells were collected and stained with annexin V-FITC/PI kit. (**A**) Apoptosis cells were determined on a Cytoflex S flow cytometer. (**B**) Percentages of apoptosis cells (early apoptotic cells and late apoptotic cells in the total cells) were presented. The values represent mean ± standard deviation of three independent experiments. Different letters indicate significant differences, *p* < 0.05.

The induction of apoptosis by PEO was evaluated using an annexin V-FITC/PI kit. As shown in Figure 4, PEO treatment dramatically promoted cell apoptosis in both A549 and HepG2 cells. Compared with HepG2 cells, A549 cells were more sensitive to PEO, consistent with the CCK8 assay results. When treated with PEO at 90 μg/mL, the apoptosis rates of A549 and HepG2 cells were (42.270 ± 5.404%) and (21.127 ± 1.208%), respectively. Compared with the control groups, the A549 and HepG2 cells treated with PEO at 30 μg/mL and 60 μg/mL exhibited significantly increased rates of apoptosis, while no significant differences in apoptosis were observed between the groups treated at 30 μg/mL and 60 μg/mL. The results clearly show that PEO induced cell apoptosis in these two cell lines but did not show typical dose-dependent characteristics.

#### 2.4.2. Hoechst 33342 Staining

**Figure 5:**
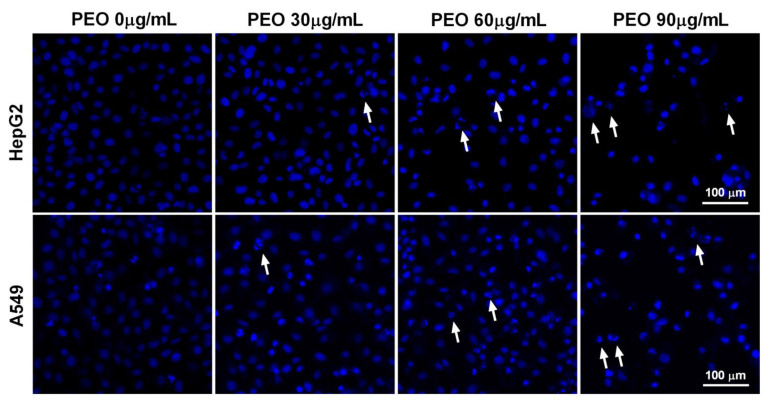
Apoptosis-inducing effect of PEO in HepG2 and A549 cells stained with Hoechst 33342. HepG2 and A549 cells were treated with or without different concentrations of PEO (30, 60, and 90 μg/mL) for 24 h, then stained with Hoechst 33342 for 15 min at 37 °C. After washing twice with PBS, the cells were photographed using ImageXpress Micro Confocal High-Content Imaging System. White arrows indicate apoptotic bodies.

Hoechst 33342 staining was performed to confirm the flow cytometry results. As shown in Figure 5, the nuclei in the control group were stained light blue by Hoechst 33342. Treating HepG2 and A549 cells with PEO at 30, 60, and 90 μg/mL for 24 h resulted in dramatic changes in cell morphology that are typical of apoptosis (e.g., cell shrinkage, nuclear condensation, membrane blebbing, and formed apoptotic body formation) [23]. Cells treated with PEO, especially at the dose of 90 μg/mL, displayed strong blue fluorescence, consistent with the flow cytometry results. These findings suggest that the cytotoxicity of PEO is mainly due to its ability to induce apoptosis.

### 2.5. Cell Cycle Analysis

**Figure 6:**
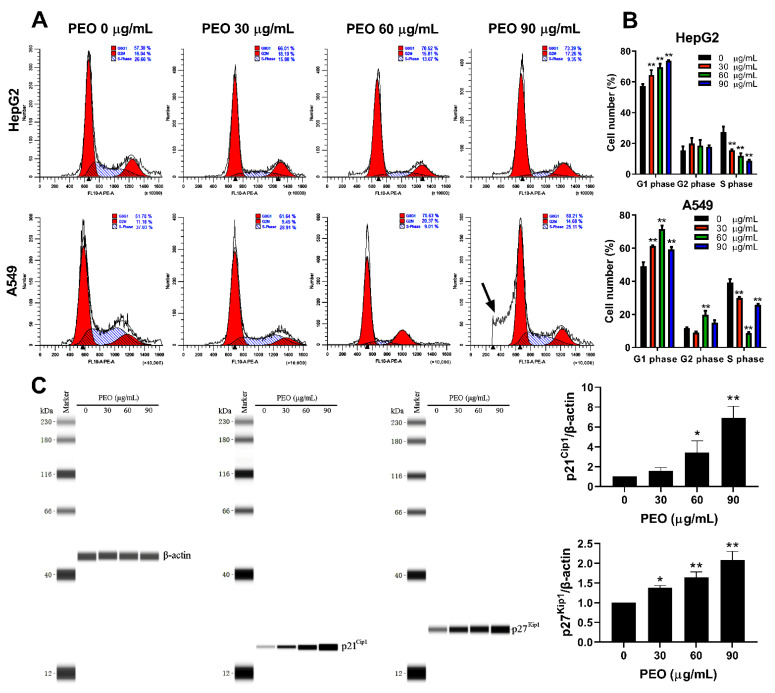
Cell cycle analysis of HepG2 and A549 cells treated with PEO. (**A**) HepG2 and A549 cells were treated with or without PEO (30, 60, and 90 µg/mL) for 24 h, and then cells were collected and fixed overnight. Cells were centrifugated and resuspended in PBS with PI/RNase Staining Buffer for 30 min. The cell cycle distributions were analyzed on a Cytoflex S flow cytometer. (**B**) Percentage of first gap (G1), synthesis (S), second gap (G2) phase cells in the total cells. (**C**) Expression levels of p21^Cip1^ and p27^Kip1^ in A549 cells treated with PEO as detected by western blot. The values represent mean ± standard deviation of three independent experiments. * *p* < 0.05, ** *p* < 0.01 compared with the untreated group. The black arrow indicates cell debris.

Cell cycle arrest can lead to the inhibition of cell growth. Therefore, the cell cycle was analyzed after PEO treatment was analyzed. As shown in Figure 6A,B, PEO treatment dramatically changed the cycle progression of HepG2 cells in a concentration-dependent manner. When cells were treated with increasing concentrations of PEO (30, 60, and 90 µg/mL) for 24 h, the percentage of S phase cells in HepG2 cells decreased from (27.237 ± 3.739%) to (8.627 ± 0.735%), while the percentage of G1 phase cells increased from (57.220 ± 1.251%) to (73.607 ± 0.474%). The cell cycle progression of A549 cells also changed significantly after PEO treatment. Similar to the HepG2 cells, the percentage of A549 cells in the G1 phase also increased significantly after PEO treatment, with the highest percentage (71.533 ± 2.013%) observed at 60 μg/mL. The change in the percentage of cells in the S phase was opposite the change observed in the percentage of G1 phase cells. The decrease in the percentage of G1 phase cells when the PEO treatment concentration increased from 60 to 90 μg/mL may be related to the induction of a large amount of cell apoptosis, which was verified by the appearance of a large amount of cell debris (indicated by the black arrow in Figure 6A). These results confirm the ability of PEO to induce cell apoptosis. Obviously, the increase of the G1 peak indicates that PEO can inhibit the proliferation of HepG2 and A549 cells by blocking the G1 phase. It is well known that cyclin kinase inhibitor proteins p21^Cip1^ and p27^Kip1^ play a pivotal role in the regulation of the G1 phase [24]. Therefore, the expression levels of p21^Cip1^ and p27^Kip1^ in PEO-treated A549 cells (which are more sensitive than HepG2 cells) were quantified using western blot. As depicted in Figure 6C, treatment with PEO increased the expression of p21^Cip1^ and p27^Kip1^ in a dose-dependent manner, implying that PEO exerts its G1 arrest effect by upregulating the expression of p21^Cip1^ and p27^Kip1^.

### 2.6. PEO Treatment Decreased Mitochondrial Membrane Potential in HepG2 and A549 Cells

**Figure 7:**
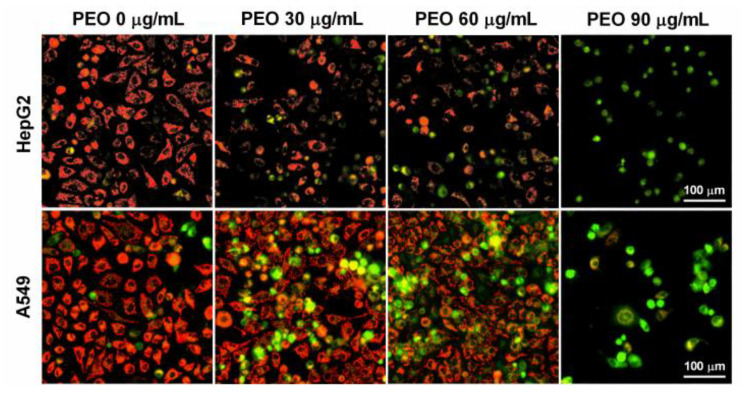
Mitochondrial membrane potential staining of HepG2 and A549 cells treated by PEO. HepG2 and A549 cells were treated with or without different concentrations of PEO (30, 60, and 90 μg/mL) for 24 h, then stained with JC-1 for 20 min at 37 °C. After washing twice with PBS, the cells were photographed using ImageXpress Micro Confocal High-Content Imaging System.

Mitochondria are not only the powerhouses of the cell, they are also important dynamic signaling organelles that control the cell cycle, including proliferation, survival, and death [25]. The loss of mitochondrial membrane potential will lead to cell apoptosis [25]. Therefore, JC-1 staining was performed to examine whether PEO induced apoptosis in cancer cells by interfering with the mitochondrial membrane potential. The results displayed that the control cells emitted strong red fluorescence, indicating that the JC-1 dye accumulated in the mitochondria of normal cells (Figure 7). In contrast, in both A549 and HepG2 cells, PEO treatment significantly reduced the number and fluorescence intensity of red fluorescent cells, while PEO treatment significantly increased the number and fluorescence intensity of orange and green fluorescent cells. When treated with PEO at 90 μg/mL, most cells emitted primarily green fluorescence. These results clearly demonstrated that the mitochondrial membrane potential was lost in PEO-treated cells, further confirming that PEO induced apoptosis in A549 and HepG2 cells apoptosis.

### 2.7. PEO Treatment Reduced ROS Levels in HepG2 and A549 Cells

**Figure 8:**
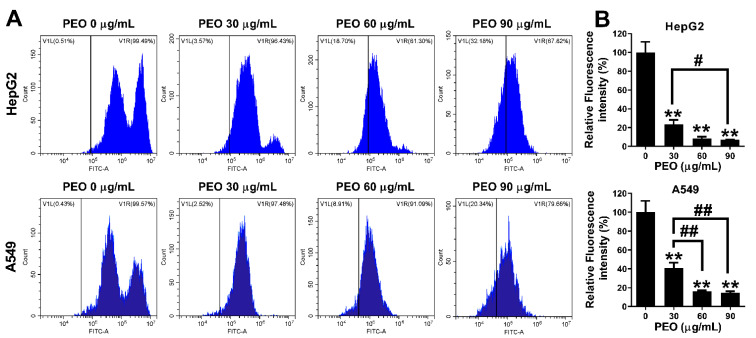
ROS levels of HepG2 and A549 cells treated by PEO. HepG2 and A549 cells were treated with or without different concentrations of PEO (30, 60 and 90 μg/mL) for 24 h, then cells were collected and stained with DCFH-DA. (**A**) Fluorescence intensity was recorded on a Beckman Cytoflex S flow cytometer. (**B**) Relative fluorescence intensity was calculated; the values represent mean ± standard deviation of three independent experiments. ^#^ *p* < 0.05, ^##^ *p* < 0.01, comparison between the two groups; ** *p* < 0.01 Compared with the control group.

Many chemotherapeutic drugs promote the production of excess ROS, which induces mitochondrial membrane depolarization and leads to cancer cell apoptosis [26]. To determine whether the loss of mitochondrial membrane potential in this study was related to the excessive production of ROS induced by PEO, the levels of ROS in cells were measured using the DCFH-DA method. As shown in Figure 8, both HepG2 and A549 cells showed high levels of ROS, which was consistent with a report that cancer cells have inherently high ROS levels compared to their normal counterparts [27]. Surprisingly, in the present study, PEO treatment not only did not increase the level of ROS, in fact, the PEO treatment significantly reduced the production of ROS. When treated with PEO at 30 μg/mL, the levels of ROS in HepG2 and A549 cells decreased to 21.134% and 13.513% of the control group, respectively. In general, PEO inhibited ROS production in a dose-dependent manner, although no significant difference in ROS level was observed between the group treated with PEO at 60 μg/mL and 90 μg/mL. Antioxidant agents can also inhibit the growth of tumor cells [27]. However, the ROS levels of cells treated with PEO at 60 μg/mL and 90 μg/mL were not significantly different, while treatment with PEO at 90 μg/mL had a remarkable proapoptotic effect. This implies that ROS inhibition was not the key molecular mechanism of PEO-induced apoptosis. Thus, other potential molecular mechanisms should be considered.

### 2.8. PEO Induces Apoptosis through a Mitochondrial Pathway

**Figure 9:**
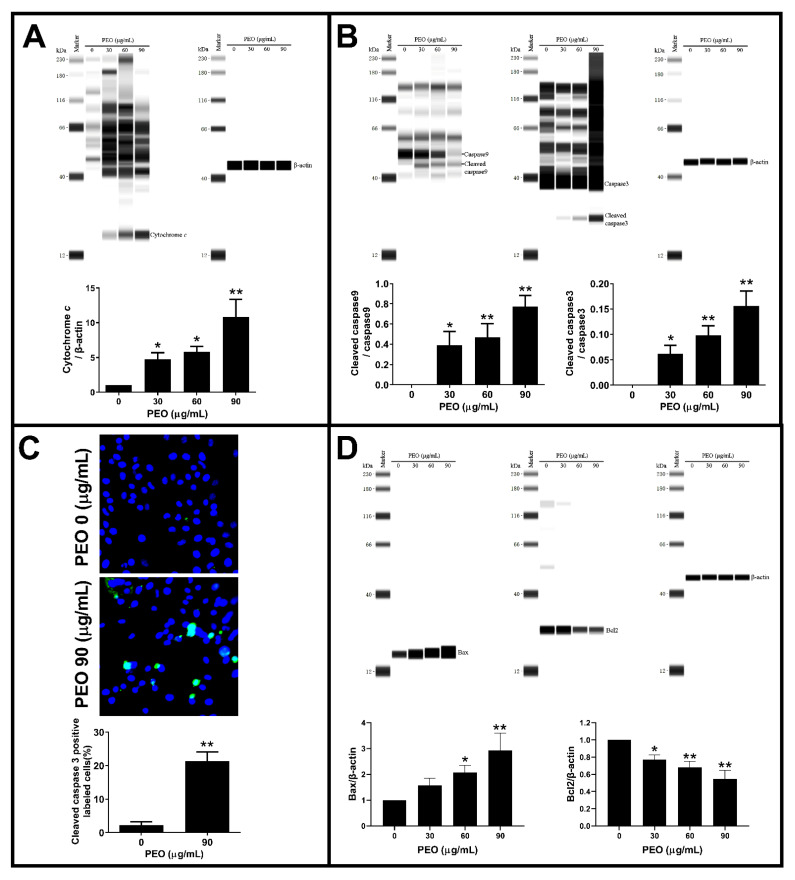
Effects of PEO on the expression of apoptosis-related proteins in A549 cells. A549 cells were treated with different concentrations of PEO for 9 h, cells were harvested, and the cytoplasmic protein was extracted with cell mitochondria isolation kit, then (**A**) the cytochrome *c* levels in cytoplasm were determined with β-actin as internal standard. Additionally, the whole cell lysates were prepared, and the protein levels of (**B**) cleaved caspase 9/caspase 9 and cleaved caspase 3/caspase 3 were detected with β-actin as internal standard. (**C**) For detection of cleaved caspase 3 in apoptotic cells, A549 cells were treated with 90 μg/mL PEO for 9 h, then cells were fixed, and permeabilized for immunocytochemistry with anti-cleaved caspase 3 primary antibody. After incubation with Alexa Fluor secondary antibody, cells were re-stained with DAPI to visualize the nucleus. The anticleaved caspase 3 positive labeled cells (green) were remarkably increased after PEO exposure. (**D**) A549 cells were treated with different concentrations of PEO for 9 h, the whole cell lysates were prepared, and the protein levels of Bax and Bcl2 were detected with β-actin as internal standard. The values represent mean ± standard deviation of three independent experiments. * *p* < 0.05, ** *p* < 0.01 compared with the control group.

The depolarization of the mitochondrial membrane potential will increase mitochondrial permeability and release cytochrome *c*, thereby activating the caspase 9/caspase 3 pathway and inducing cell apoptosis [28]. To confirm whether PEO induces apoptosis through a mitochondrial pathway, the expression level of proteins related to mitochondrially mediated apoptosis was detected. In general, PEO showed similar inhibitory and apoptotic effects on HepG2 and A549 cells, with A549 cells being more sensitive to PEO than HepG2 cells. Therefore, A549 cells were used to explore the possible molecular mechanism of PEO-induced apoptosis. As shown in Figure 9A, PEO exposure increased the amount of cytosolic cytochrome *c* in A549 cells in a dose-dependent manner with the highest level of cytochrome *c* in cytoplasm observed after treatment with PEO at 90 µg/mL. The release of cytochrome *c* from mitochondria activates caspase 9 and caspase 3 and leads to cell apoptosis [29]. Therefore, the proteins level of caspase 9, cleaved caspase 9, caspase 3, and cleaved caspase 3 were determined. As manifested in Figure 9B, PEO stimulation dose-dependently increased the ratio of cleaved caspase 9/caspase 9 and cleaved caspase 3/caspase 3, which was further confirmed by the detection of the dramatically increased of cleaved caspase 3 positive cells after PEO (90 μg/mL) exposure (Figure 9C).

Multiple factors can lead to cytochrome *c* release from mitochondria, such as oxidative stress, imbalance of B-cell lymphoma-2 (Bcl-2) family proteins, and disturbance of cellular Ca^2+^ homeostasis [30]. In this study, we did not observe an increased ROS in PEO-treated HepG2 and A549 cells. On the contrary, PEO application can significantly attenuate the intracellular ROS level in a dose-dependent manner (Figure 8). Bcl-2 family proteins play a critical role in the regulation of mitochondrial cytochrome *c* release. Bcl-2 is an apoptosis-preventing protein, the effect of which can be counteracted by the proapoptotic protein Bcl-2-associated X protein (Bax). The increase of the Bax/Bcl-2 ratio can promote the release of cytochrome *c* from mitochondria, which activates caspase 9 and caspase 3 and leads to cell apoptosis [31]. Therefore, the protein levels of Bcl-2 and Bax were determined. As depicted in Figure 9D, PEO treatment dose-dependently decreased the level of Bcl-2 while significantly increasing the level of Bax, resulting in a dramatic increase in Bax/Bcl-2 ratio. Obviously, this result was consistent with the elevated level of cytochrome *c* in the cytosol (Figure 9A). Overall, these data added support to the notion that PEO induces apoptosis through the mitochondrion-mediated intrinsic pathway.

### 2.9. Dolabella-3,7-dien-18-ol (DDO) Is One of the Main Active Components in PEO

#### 2.9.1. DDO Induces Apoptosis through a Mitochondrial Pathway

**Figure 10:**
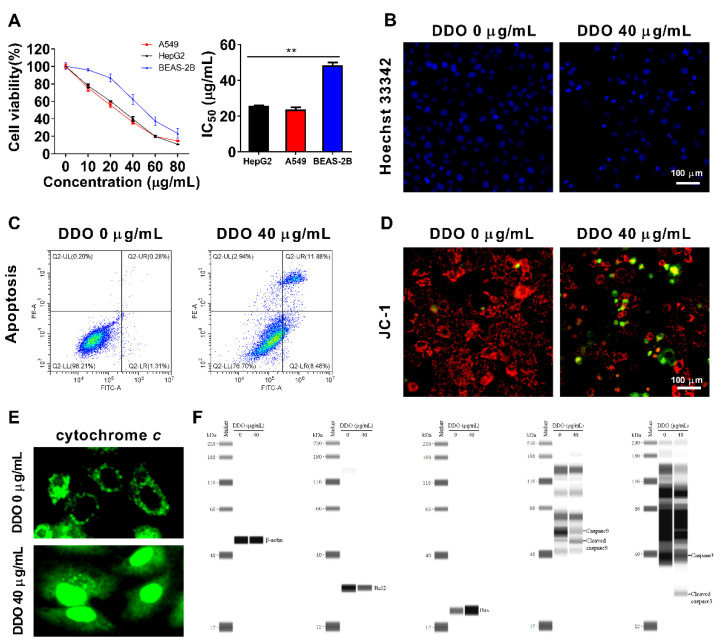
Cytotoxicity and apoptosis-inducing effect of compound DDO. HepG2, A549, and BEAS-2B cells were treated with or without a series concentration of DDO for 24 h, then cell viability was determined by (**A**) CCK8 assay and IC_50_ was calculated; the values represent mean ± standard deviation of three independent experiments. A549 cells were treated with DDO (40 µg/mL) for 24 h, apoptosis cells were stained with (**B**) Hoechst 33342 and (**C**) annexin V-FITC/PI kit, and detected with ImageXpress Micro Confocal High-Content Imaging System and flow cytometry, respectively. (**D**) After being treated with DDO (40 µg/mL) for 24 h, A549 cells were stained with JC-1 to observe the mitochondrial membrane potential. A549 cells were treated with DDO (40 µg/mL) for 9 h, (**E**) a part of cells was used for immunofluorescence analysis of the release of cytochrome *c* from mitochondria, (**F**) and another part of cells was harvested to prepare total proteins, which were then subjected to Simple Wes System for detection of apoptosis-related proteins. The values represent mean ± standard deviation of three independent experiments, ** *p* < 0.01.

We examined if the antitumor activity displayed by PEO could be attributed to the high content of DDO. As shown in Figure 10A, DDO inhibited cell growth in both HepG2 and A549 cells in a concentration-dependent manner with IC_50_ values of (25.820 ± 0.216) µg/mL and (23.597 ± 1.207) µg/mL, respectively, which were significantly lower than the IC_50_ value (48.253 ± 1.677 µg/mL) of the normal human bronchial epithelial cell line BEAS-2B. In general, DDO showed similar effects on HepG2 and A549 cells in terms of cell growth inhibition (Figure 10A), apoptosis, and mitochondrial membrane potential (Figure S2). Therefore, the following experiments were performed on A549 cells to explore the possible molecular mechanism of DDO. As depicted in Figure 10B, Hoechst 33342 staining further confirmed the inhibitory effect of DDO on cell growth. The number of cells treated with 40 µg/mL of DDO decreased dramatically, the cell morphology became smaller and round, the nucleus concentrated, and the staining deepened, showing typical apoptotic characteristics, which was reconfirmed by flow cytometry (Figure 10C). Moreover, the cell mitochondrial membrane potential was detected using the JC-1 staining method. As shown in Figure 10D, the mitochondria of the control group emitted strong red fluorescence after JC-1 staining, but many cells treated with 40 µg/mL DDO emitted green fluorescence, indicating the depolarization of mitochondrial membrane potential. Consequently, the depolarization of the mitochondrial membrane led to the release of a large amount of cytochrome *c* from the mitochondria into the cytosol (Figure 10E), which in turn activated the caspase 9/caspase 3 pathway (Figure 10F) and resultant cell apoptosis. The depolarization of mitochondrial membrane potential may be largely attributed to the upregulation of Bax and the downregulation of Bcl2 by DDO (Figure 10F). Obviously, consistent with PEO, DDO can also induce cell apoptosis through a mitochondrial-mediated intrinsic pathway.

#### 2.9.2. DDO Induces G1 Phase Arrest by Upregulating the Expression of p21^Cip1^

**Figure 11:**
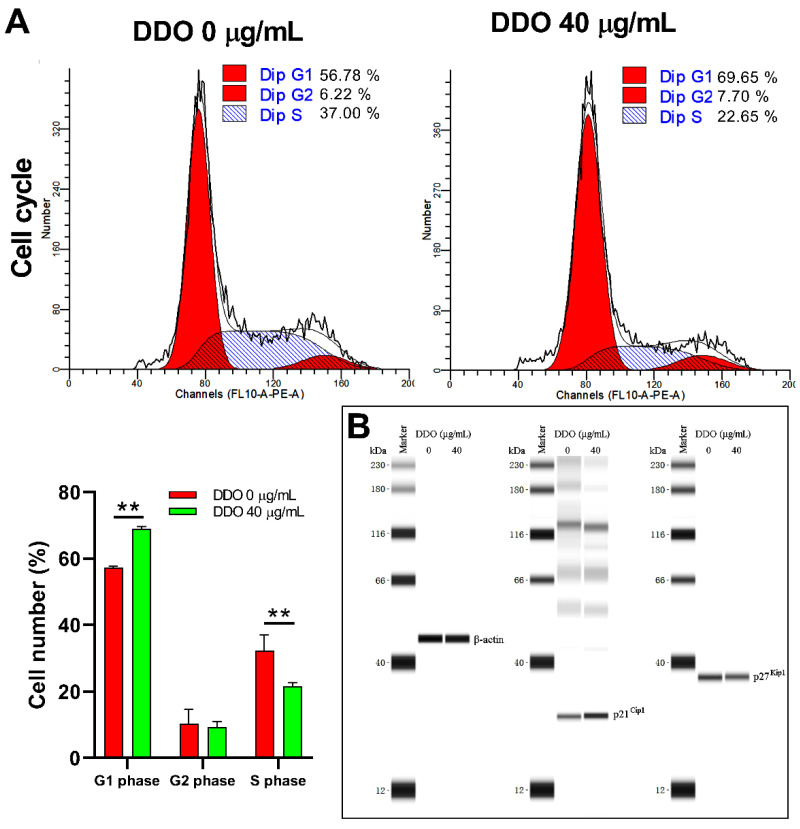
Cell cycle analysis of A549 cells treated with DDO. (**A**) A549 cells were treated with DDO (40 µg/mL) for 24 h; cells were fixed and stained with PI/RNase Staining Buffer for 30 min. The cell cycle distributions were analyzed on a Cytoflex S flow cytometer. (**B**) Expression levels of p21^Cip1^ and p27^Kip1^ in A549 cells treated with DDO (40 µg/mL) were detected on a Simple Wes System. The values represent mean ± standard deviation of three independent experiments. ** *p* < 0.01.

Considering the significant cell cycle arrest effect of PEO, the effect of DDO on the cell cycle was evaluated. The results showed that 40 µg/mL DDO treatment also dramatically induced G1 phase arrest of A549 cells (Figure 11A). However, its molecular mechanism was not completely consistent with PEO. DDO 40 µg/mL significantly upregulated p21^Cip1^ expression but had no significant effect on the p27^Kip1^ protein level (Figure 11B).

The above effects of DDO were basically consistent with PEO, implying that DDO was one of the main functional components of PEO to inhibit tumor cell growth and induce apoptosis.

### 2.10. Potential Molecular Mechanisms of PEO and DDO against A549 Cells

**Figure 12:**
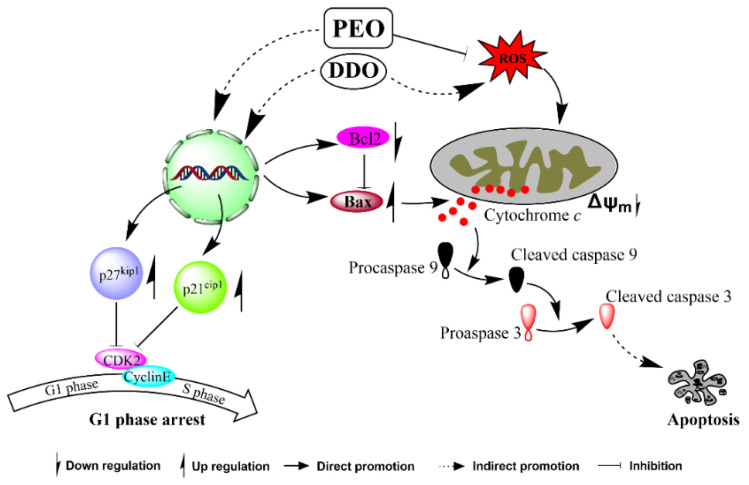
The possible mechanisms for PEO- and DDO-induced G1 phase arrest through upregulating p21^cip1^ and p27^kip1^ signaling and apoptosis via mitochondria-dependent pathway in A549 cells.

Antitumor active components often play an antitumor role through a variety of molecular mechanisms [32,33]. The present study demonstrated that both active compound DDO and essential oil PEO showed remarkable cell cycle arrest and apoptosis. It is well known that the cell cycle is driven by sequential activation of the cyclin-dependent kinase (Cdk)/cyclin complex. The activity of Cdk is negatively regulated by the Cip/Kip protein family, such as p21^Cip1^ and p27^Kip1^, which inhibit the activity of G1/S kinases, leading to cell G1 phase arrest [34,35]. PEO treatment significantly induced G1 phase arrest in A549 cells. Western blotting showed that the expression of p21^Cip1^ and p27^Kip1^ was positively correlated with the dosage of PEO, which revealed that PEO could inhibit the growth of A549 cells, at least partially, by increasing the levels of p21^Cip1^ and p27^Kip1^ proteins (Figure 12). The active compound DDO can also significantly block A549 cells in the G1 phase by upregulating p21^Cip1^ rather than p27^Kip1^ at 40 µg/mL, implying that some other active components in PEO may participate in G1 phase blocking.

In addition to regulating the cell cycle, p21^Cip1^ and p27^Kip1^ also play an important role in regulating apoptosis [36,37]. In a variety of cancer cells, such as prostate cancer cells [38], cervical cancer cells [39], gastric cancer cells [40], and lung cancer cells [41], overexpression of p21^Cip1^ or p27^Kip1^ leads to apoptosis. In addition, many active components can also induce apoptosis of cancer cells by upregulating p21^Cip1^ and p27^Kip1^ [42,43,44]. However, growing evidence also suggests that p21^Cip1^ or p27^Kip1^ can promote tumors and enhance their resistance to apoptosis, indicating a paradoxical effect [36,45]. The proapoptotic and antiapoptotic effects of p21^Cip1^ and p27^Kip1^ are largely related to their different phosphorylation sites and subcellular localization [46]. Therefore, whether PEO and DDO affect p21^Cip1^ and p27^Kip1^ phosphorylation and subcellular localization remains to be further studied. It is well known that the increase of the Bax/Bcl2 ratio can enhance the permeability of the mitochondrial membrane and promote the release of cytochrome *c* from mitochondria to cytosol, thus activating caspase cascade (Figure 12) to initiate the mitochondrial-mediated intrinsic apoptosis pathway [31]. In the present study, Annexin V-FITC/PI staining flow cytometry, Hoechst 33342 staining microscopic analysis, JC-1 staining mitochondrial membrane potential measurement, immunofluorescence detection, and western blot analysis conjointly provided solid evidence for PEO- and DDO-induced A549 cell apoptosis (Figure 12).

Oxidative stress is another causal factor of mitochondrial membrane potential depletion and apoptosis [26]. Compared with the control group, the ROS level in A549 cells increased by about 1.4 times after DDO (40 µg/mL) exposure (see Figure S3). However, the ROS level of the A549 cells was significantly attenuated in a dose-dependent manner after PEO treatment, with 86.587% ROS elimination at peak dose (Figure 8). Given DDO accounts for 25.5% of PEO and both of them exert an apoptotic effect, it is reasonable to postulate that the mitochondrial intrinsic pathway rather than the ROS could represent the mechanism underlying DDO-induced apoptosis.

## 3. Materials and Methods

### 3.1. Plant Material

Whole *P. acutum* plants were collected from the forest in Hangzhou (30°16′ N; 120°15′ E; 11.0 m a.s.l.), Zhejiang, China, in June 2021. The species was identified by Dr. Zhi-Shan Ding, School of Medical Technology and Information Engineering, Zhejiang Chinese Medical University, Hangzhou, China. A voucher specimen (No. 2021-06-06) was deposited at the School of Life Sciences, Zhejiang Chinese Medical University.

### 3.2. Simultaneous Distillation–Extraction (SDE) of Essential Oil

**Figure 13:**
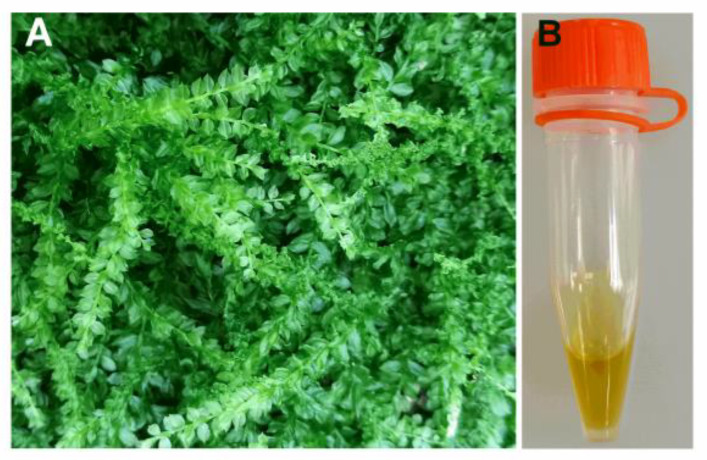
Morphological observation of the aerial parts of (**A**) *P*. *acutum* and (**B**) essential oil extracted by SDE method.

The fresh aerial parts of the *P. acutum* plants were collected (Figure 13A), and the other plant materials and soil were removed to avoid contamination. The fresh sample (600 g) was homogenized in 3 L of water and extracted by SDE in a classical Likens–Nickerson apparatus for 4 h with 100 mL of dichloromethane. The organic phase was collected and dried with anhydrous MgSO_4_ and concentrated at 25 °C under reduced pressure to obtain a light-yellow essential oil (Figure 13B).

### 3.3. Chemical Composition

#### 3.3.1. GC/MS Analysis of *P. acutum* Essential Oil (PEO)

The components of PEO were characterized using a gas chromatograph (Agilent Technologies 7890B) equipped with a mass-selective detector and analyzer (Agilent 7250) with time-of-flight electrospray ionization (Agilent Technologies, Chicopee, MA, USA) and an HP-5 capillary column (30 m × 0.25 mm i.d.; film thickness = 0.25 μm). After maintaining the initial column temperature of 50 °C for 3 min, the temperature was increased to 280 °C at 5 °C/min and held for 5 min. The temperature of the injector was maintained at 250 °C. The carrier gas was helium with a flow rate of 1.0 mL/min. The sample (1 μL diluted with n-hexane) was injected at a split ratio of 10:1. The mass spectra were detected in the *m*/*z* range of 40–450 with an electron energy of 70 eV. The temperatures of the quad and ion source were maintained at 150 °C and 250 °C, respectively.

#### 3.3.2. Identification of Individual Components

The PEO components were recognized by comparison with their Kovats retention index (RI) relative to the obtained C_7_–C_40_ n-alkanes series (O2si, North Charleston, SC, USA) on an HP-5MS column; calculation of RI and comparison with reported in the literature and National Institute of Standards and Technology (NIST) Standard Reference Database (NIST Chemistry WebBook, 2014) as well as by comparison of mass spectra with those registered by the National Institute of Standards and Technology (NIST 14). Each PEO component was determined by RI and compared with the known components reported previously [8]. The contents of the PEO components were calculated from the GC/MS peak areas without any correction factor. An uncertain compound was purified by silica gel chromatography and identified by nuclear magnetic resonance (NMR) spectroscopy (Figure S1).

### 3.4. In Vitro Antitumors Assay

#### 3.4.1. Cytotoxicity Analysis

The cytotoxic effects of PEO against the BEAS-2B, MCF-7, HepG2, A549, and U87 cell lines, which were obtained from the Institute of Biochemistry and Cell Biology, China Academy of Sciences, were evaluated in vitro using CCK8 (Biosharp, Hefei, China) assay. Curcumin was used as a positive control. Cells in the exponential growth phase were inoculated into 96-well plates at an initial density of 8000 cells per well and treated with the indicated concentrations of PEO for 24 h in triplicate, and then 10 μL of CCK8 was added to each well. After incubation for 4 h at 37 °C, the absorbance was measured on a microplate reader (Perkin Elmer, Enspire, Singapore) at 450 nm. The IC_50_ value was calculated as the concentration of drug yielding a 50% cell survival rate based on the measured absorbance.

#### 3.4.2. RTCA

To detect the effect of PEO on the proliferation of cancer cells in real-time, cells were treated with different concentrations of PEO and monitored using an xCELLigence real-time cell analyzer (DP system, ACEA Biosciences, San Diego, CA, USA). Briefly, after the E-Plate96 (ACEA Biosciences) was pretreated with 50 μL of culture medium, 5000 cells in 100 μL of culture medium were seeded into the E-Plate96. The E-Plate96 was then locked in the RTCA device at 37 °C with 5% CO_2_. At 20 h after plating, the cells were treated with different concentrations of PEO. The cell index (CI) was determined as the measured changes in electrical impedance, which directly reflects cellular proliferation on biocompatible microelectrode-coated surfaces. CI was read automatically every 15 min, and the recorded curves were normalized to the time point of cell seeding, referred to as normalized CI. All experiments were performed in triplicate.

#### 3.4.3. Cell Cycle Analysis

Cell cycle assay was performed as described previously [47]. Cells in the exponential growth phase were seeded in a six-well plate at a density of 2.0 × 10^5^ cells/well and allowed to attach overnight. The cells were then treated with the indicated concentration of PEO (30, 60, and 90 μg/mL) or DDO (40 μg/mL) for 24 h. The cells were washed three times with ice-cold PBS (pH 7.4) and fixed overnight with 70% ethanol at 4 °C followed by centrifugation at 300× *g* for 8 min. The cells were resuspended in PBS (pH 7.4) with PI/RNase staining buffer (BD, 550825, San Diego, CA, USA) for 30 min. The cell cycle distributions were analyzed using a Cytoflex S flow cytometer (Beckman, Brea, CA, USA).

#### 3.4.4. Apoptosis Analysis

Following the procedure of Mao et al. [48], an annexin V-FITC/PI apoptosis kit (BD Biosciences) was used to determine the extent of apoptosis and necrosis in response to PEO exposure. Cells were seeded in a six-well plate at a density of 2.0 × 10^5^ cells/well and incubated at 37 °C for the indicated times in the presence or absence of various concentrations of PEO (30, 60, and 90 μg/mL) or DDO (40 μg/mL). The cells were then trypsinized, pelleted, washed in ice-cold PBS (pH 7.4), and resuspended in 1× binding buffer according to the manufacturer’s instructions. Subsequently, the cells were incubated with annexin V-FITC and PI for 15 min at room temperature in the dark. The stained cells were analyzed using a Cytoflex S flow cytometer (Beckman, Brea, CA, USA).

#### 3.4.5. Hoechst 33342 Staining

Hoechst 33342 (C1022, Beyotime, Haimen, China) staining was performed using a fluorescence microscope as described by Shang et al. [49]. After seeding cells at a density of 8000 cells/well in a 96-well plate and allowing the cells to attach overnight, the cells were treated with different concentrations of PEO (30, 60, and 90 μg/mL) or DDO (40 μg/mL) for 24 h. The cells were then washed three times with PBS (pH 7.4) and stained with 5 μg/mL of Hoechst 33342 for 15 min at 37 °C in the dark. After washing twice with PBS, the cells were photographed using an ImageXpress Micro Confocal High-Content Imaging System (Molecular Devices, Sunnyvale, CA, USA).

### 3.5. Mitochondrial Membrane Potential Staining

The decreased mitochondrial membrane potentials of cells were measured using a JC-1 mitochondrial membrane potential detection kit (Beyotime, Haimen, China) according to the manufacturer’s instructions. Briefly, cells were inoculated into 96-well plates at an initial density of 8000 cells/well in triplicate and treated with the indicated concentrations of PEO or DDO for 24 h, The cells were stained with JC-1 working solution (2 μM) for 20 min at 37 °C in the dark. After washing twice with PBS, the light emissions were recorded on an ImageXpress Micro Confocal High-Content Imaging System (Molecular Devices, Sunnyvale, CA, USA).

### 3.6. Reactive Oxygen Species (ROS) Assay

The fluorescent intensity of ROS generation was determined as described by Zhang et al. [50]. Cells were seeded into six-well plates, incubated overnight, and treated with different concentrations of PEO for 24 h. The cells were then trypsinized, pelleted, and resuspended in 0.5 mL culture medium and stained with 10 mM DCFH-DA (Beyotime, Haimen, China) for 30 min at 37 °C in the dark. After rinsing twice with PBS, the fluorescence intensity was measured using a Cytoflex S flow cytometer (Beckman, Brea, CA, USA).

### 3.7. Western Blotting Analysis

Western blotting was performed as described previously [47]. Cells were collected after treatment with different concentrations of PEO (30, 60, and 90 μg/mL) or DDO (40 μg/mL) for 9 h. To determine the cytochrome *c* levels in cytoplasm, the cytoplasmic protein was extracted using a cell mitochondria isolation kit (C3601, Beyotime, Haimen, China) according to the manufacturer’s instructions. The whole-cell lysates were prepared using M-PER Mammalian Protein Extraction Reagent (78503, Thermo Fisher Scientific, Waltham, MA, USA) containing protease and phosphatase inhibitor (Roche, Germany) at 4 °C for 30 min. The samples were centrifuged at 14,000× *g* for 10 min, and the supernatants were transferred to a new tube for detection of the protein levels of cytochrome *c*, caspase 9, and caspase 3; β-actin served as the internal standard.

According to our previous report [47], the protein concentration of each sample was determined using the BCA method. Immunoblotting was performed on a Simple Wes System (ProteinSimple, San Jose, CA, USA) using a Size Separation Master Kit with Split Buffer (12–230 kDa) according to the manufacturer’s instructions with anti-Bax (2772T, CST, Danvers, MA, USA), Bcl-2 (3498T, CST, Danvers, MA, USA), cytochrome *c* (A4912, ABclonal, Wuhan, China), caspase 9 (66169-1-Ig, proteintech, Chicago, IL, USA), caspase 3 (66470-2-Ig, proteintech, Chicago, IL, USA), and anti-β-actin (4967S, CST, Danvers, MA, USA) antibodies. Compass software (version 6.0.0, ProteinSimple) was used to program the Simple Wes and to present (and quantify) the western immunoblots. The output data displayed by the software were the averages of seven exposures (5–480 s).

### 3.8. Immunofluorescence Analysis

A549 cells were treated with 90 μg/mL PEO or 40 μg/mL DDO for 9 h, fixed with 4% formaldehyde for 20 min, permeabilized with 0.1% TritonX-100 for 15 min, washed three times with PBS, and blocked with 10% FBS in PBS for 1h at room temperature. The cells were then stained with an anti-cleaved caspase 3 (9664, CST, Danvers, MA, USA) or anti-cytochrome *c* (A4912, ABclonal, Wuhan, China) antibody diluted at 1:400 in 10% FBS in PBS for 2 h at room temperature, washed three times with PBS, and incubated with donkey polyclonal secondary antibody to rabbit IgG-H&L (Alexa Fluor^®^ 488) (ab150073, Abcam, Waltham, MA, USA) diluted at 1:1000 in 1% FBS in PBS for 2 h at room temperature in the dark. Finally, the nuclei were stained with DAPI (100 ng/mL), and images were collected using an ImageXpress Micro Confocal High-Content Imaging System (Molecular Devices, Sunnyvale, CA, USA).

### 3.9. Statistical Analysis

Data are presented as the mean ± standard deviation derived from at least three independent experiments. Analysis of variance was used to examine the statistical significance of the differences between the groups with *p* < 0.05 indicating significance for all the experiments.

## 4. Conclusions

To the best of our knowledge, this is the first report on the pharmacological activities of PEO. Based on GC/MS, the present study demonstrated that PEO was rich in diterpenes (26.5%), sesquiterpenes (23.89%), and alcohols (21.81%), and two compounds (selina-6-en-4-ol and dolabella-3,7-dien-18-ol) were identified in the essential oil of *P. acutum* for the first time. Dolabella-3,7-dien-18-ol, which accounted for 25.5% of the total PEO peak area, showed significant antitumor activity and was one of the main antitumor active components of PEO. PEO can dramatically block the cell cycle of A549 and HepG2 in the G1 phase, which is partially attributed to the upregulation of p21^Cip1^ and p27^Kip1^ proteins. In addition, PEO also showed a remarkable apoptosis-inducing effect in HepG2 and A549 cells, and this effect involved a mitochondrial pathway. However, PEO did not interfere with the mitochondrial membrane potential by promoting ROS burst. In contrast, PEO significantly inhibited ROS production. Thus, additional research is needed to investigate the biochemical mechanisms of PEO. The results support the use of *P. acutum* as a traditional Chinese medicine in the treatment of cancer. The findings are also helpful to guide searches for new potential antitumor agents.

## Figures and Tables

**Table 1:** Composition and content of PEO.

Class	Component	Molecular Formula	RI ^1^	RI (NIST) ^2^	RI Range ^3^	Area %
	β-Myrcene	C_10_H_16_	991	991 ± 2	980–995	0.06
	β-Ocimene, (E)-	C_10_H_16_	1049	1049 ± 2	1038–1056	0.05
	Linalool	C_10_H_18_O	1099	1099 ± 2	1088–1109	0.37
	Limonene	C_10_H_16_	1028		1027–1032	0.25
	γ-Terpinene	C_10_H_16_	1059	1060 ± 3	1049–1069	0.11
	Terpinen-4-ol	C_10_H_18_O	1178	1182 ± 0	1165–1189	0.18
	α-Terpineol	C_10_H_18_O	1191	1189 ± 2	1178–1203	0.28
	α-Pinene	C_10_H_16_	933		921–944	0.16
	Camphene	C_10_H_16_	948	952 ± 2	936–959	0.09
	β-Pinene	C_10_H_16_	976	979 ± 2	964–988	0.11
	δ-4-Carene	C_10_H_16_	1017	1009 ± 7		0.04
	Eucalyptol	C_10_H_18_O	1031	1032 ± 2		1.38
	2-Bornanone	C_10_H_16_O	1146	1143 ± 9		0.11
	β-Nerolidol	C_15_H_26_O	1565	1564 ± 2	1560–1565	1.46
	β-Elemene	C_15_H_24_	1395	1391 ± 2	1374–1402	0.1
	γ-Elemene	C_15_H_24_	1430	1433 ± 3	1410–1486	0.96
	α-Caryophyllene	C_15_H_24_	1458	1454 ± 3		0.24
	Germacrene D	C_15_H_24_	1486	1481 ± 3	1464–1493	0.38
	Acoradiene	C_15_H_24_	1469	1471 ± 8	1447–1478	0.88
	4-epi-α-acoradiene	C_15_H_24_	1479	1475		0.94
	β-Selinene	C_15_H_24_	1491	1486 ± 3	1473–1496	0.31
	δ-Selinene	C_15_H_24_	1495	1493 ± 4		0.46
	δ-Cadinene	C_15_H_24_	1527	1524 ± 2	1508–1539	0.2
	α-Calacorene	C_15_H_20_	1548	1542 ± 3	1522–1549	0.01
	Selina-6-en-4-ol	C_15_H_26_O	1624			3.82
	α-Cadinol	C_15_H_26_O	1660	1653 ± 2	1635–1664	0.34
	Drimenol	C_15_H_26_O	1769	1761 ± 11		0.23
	α-Copaene	C_15_H_24_	1379	1376 ± 2	1363–1391	0.14
	α-Cedrene	C_15_H_24_	1417	1411 ± 3	1397–1435	0.1
	β-Cedrene	C_15_H_24_	1426	1421 ± 3	1415–1434	11.39
	Aromandendrene	C_15_H_24_	1444	1440 ± 1		0.1
	β-Copaene	C_15_H_24_	1456		1426–1449	0.27
	Ledene	C_15_H_24_	1500	1493 ± 4		0.64
	Spathulenol	C_15_H_24_O	1583	1577 ± 5	1562–1590	0.57
	Globulol	C_15_H_26_O	1589	1583 ± 3	1568–1592	0.35
	Dolabella-3,7-dien-18-ol	C_20_H_34_O	2133	2162		25.5
	bifomen	C_20_H_32_	1938	1931		1
	Hexanal	C_6_H_12_O	802	800 ± 2	782–810	0.34
	Heptanal	C_7_H_14_O	903	901 ± 2	894–913	0.23
	3-Octanone	C_8_H_16_O	987	986 ± 3	971–994	1.07
	Octanal	C_8_H_16_O	1004	1003 ± 2	993–1012	0.22
	Benzeneacetaldehyde	C_8_H_8_O	1044	1045 ± 4	1032–1063	0.04
	Nonanal	C_9_H_18_O	1104	1104 ± 2	1093–1118	0.64
	2-Nonenal, (E)-	C_9_H_16_O	1160	1162 ± 3	1142–1151	0.09
	Decanal	C_10_H_20_O	1205	1206 ± 2	1195–1217	0.25
	Undecanal	C_11_H_22_O	1308	1307 ± 2	1295–1319	0.09
	Dodecanal	C_12_H_24_O	1409	1409 ± 4	1397–1420	0.22
	Tetradecanal	C_14_H_28_O	1613	1613 ± 2	1609–1615	0.21
	Pentadecanal	C_15_H_30_O	1715	1715 ± 3		0.69
	Hexadecanal	C_16_H_32_O	1817	1817 ± 6		0.34
	Nonanoic acid	C_9_H_18_O_2_	1266	1273 ± 7	1260–1293	0.09
	n-Hexadecanoic acid	C_16_H_32_O_2_	1959	1968 ± 7		1.05
	3-Hexen-1-ol, (E)-	C_6_H_12_O	855	852 ± 3	837–863	0.07
	1-Hexanol	C_6_H_14_O	868	868 ± 4	859–885	0.22
	1-Heptanol	C_7_H_16_O	970	970 ± 2	945–980	0.09
	1-Octen-3-ol	C_8_H_16_O	979	980 ± 2	967–991	14.17
	3-Octanol	C_8_H_18_O	996	994 ± 3	981–1005	5.68
	2-Octen-1-ol, (Z)-	C_8_H_16_O	1068	1067 ± 4	1059–1071	0.38
	1-Octyn-3-ol	C_8_H_14_O	1071			0.15
	Phenylethyl Alcohol	C_8_H_10_O	1112	1116 ± 5	1100–1129	0.07
	3-Nonen-1-ol, (Z)-	C_9_H_18_O	1154	1156 ± 3		0.17
	1-Nonanol	C_9_H_20_O	1171	1173 ± 2	1167–1184	0.13
	10-Undecen-1-ol	C_11_H_22_O	1678	1664		0.15
	Phytol	C_20_H_40_O	2114	2114 ± 5	2110–2122	0.53
	Ethylbenzene	C_8_H_10_	860	855 ± 10		0.03
	p-Xylene	C_8_H_10_	868	865 ± 7		0.11
	2-Hexene, 3,5,5-trimethyl-	C_9_H_18_	973	985 ± 4		0.65
	Dodecane	C_12_H_26_	1200	1200		0.07
	Tetradecane	C_14_H_30_	1400	1400	1400	0.14
	n-Hexadecane	C_16_H_34_	1600	1600		0.26
	1-Docosene	C_22_H_44_	2194	2193 ± 2		0.36
	Safrole	C_10_H_10_O_2_	1290	1287 ± 2	1280–1294	0.16
	Methyleugenol	C_11_H_14_O_2_	1406	1402 ± 3		0.41
	Butanoic acid, ethyl ester	C_6_H_12_O_2_	804	802 ± 2		0.36

^1^ Retention index experimentally calculated based on the C_7_–C_40_ n-alkanes standard. ^2^ Retention indices were estimated using the NIST GC-RI database. Retention index taken from NIST. ^3^ Range of Kováts retention indices of the composition on column of dimethylsilicone stationary phase with 5% phenyl reported by Isidorov et al. [17].

## Data Availability

The data that support the findings of this study are available from the corresponding author upon reasonable request.

## Supplementary Materials

The following supporting information can be downloaded at: https://www.mdpi.com/article/10.3390/ijms232314790/s1. Figure S1. Identification of the purified compound dolabella-3,7-dien-18-ol. Figure S2. Effects of DDO on apoptosis and mitochondrial membrane potential of HepG2 and A549 cells. Figure S3. ROS levels of A549 cells treated by DDO.
