# Supplementary material for: Chemical Composition, Antitumor Properties, and Mechanism of the Essential Oil from Plagiomnium acutum T. Kop
Source: Int J Mol Sci. 2022 Nov 26;23(23):14790. doi: 10.3390/ijms232314790 (PMC9738176; doi:10.3390/ijms232314790)
Supplement: Supplementary file 1 [file ijms-23-14790-s001.zip › ijms-1988816-supplementary.pdf]

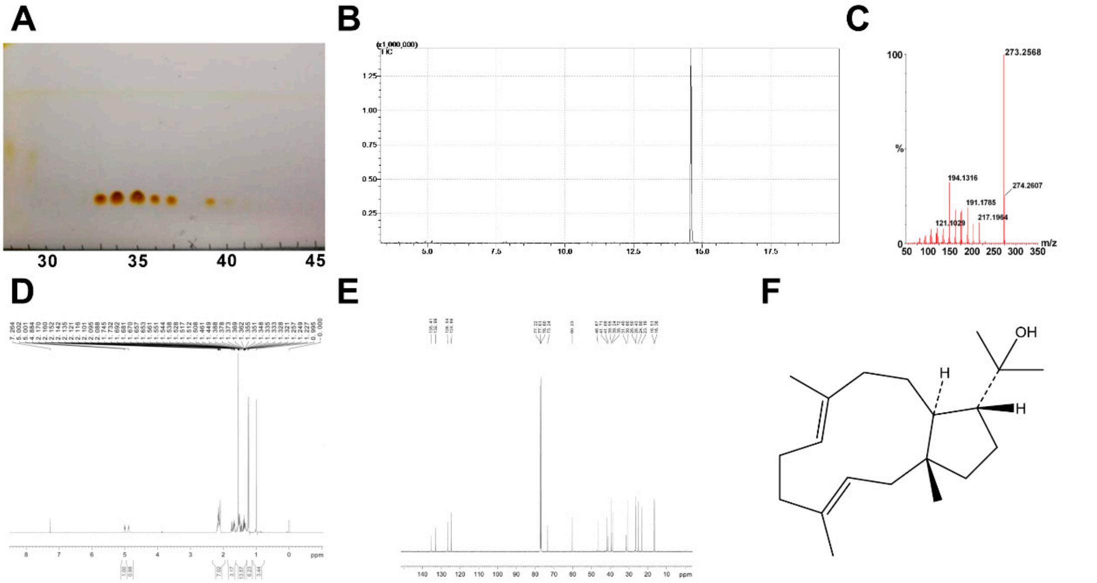


**Figure S1**. Identification of the purified compound dolabella-3,7-dien-18-ol. Two hundred and twenty milligram PEO was separated on silica gel (200–300 mesh, Qingdao Haiyang Chemical Co., Ltd., Qingdao, China) column (15 × 2 cm) chromatography and eluted with petroleum ether-ethyl acetate (20:1 *v:v*) and collected in 5 mL/tube. (**A**) Thin layer chromatography (TLC) was performed on precoated silica gel 60 F254 plates (Qingdao Haiyang Chemical Co., Ltd., Qingdao, China), developed with petroleum ether-ethyl acetate (20:1 *v:v*) and then exposed to the iodine vapor for 10 min. The target compound tubes (33–37) were combined to give 87 mg of oily substance. (**B**) The combined fraction was characterized using a gas chromatograph (Agilent Technologies 7890B) equipped with a mass-selective detector and analyzer (Agilent 7250) with time-of-flight electrospray ionization (Agilent Technologies, MA, USA), an HP-5 capillary column (30 m × 0.25 mm i.d.; film thickness = 0.25 μm). After maintaining the initial column temperature of 50°C for 3 min, the temperature was increased to 280 °C at 5 °C/min and held for 5 min. The temperature of the injector was maintained at 250 °C. The carrier gas was helium with a flow rate of 1.0 mL/min. The sample (1 μL diluted with n-hexane) was injected at a split ratio of 10:1. The mass spectra were detected in the *m*/*z* range of 40–450 with an electron energy of 70 eV. The temperatures of the quad and ion source were maintained at 150 °C and 250 °C, respectively. No obvious impurity peak was observed in the GC/MS results, therefor the purity of the target component was high. (**C**) Mass spectrogram recorded on a gas chromatograph (Agilent Technologies 7890B) equipped with a mass-selective detector and analyzer (Agilent 7250) with time-of-flight electrospray ionization (Agilent Technolo-gies, MA, USA). (**D**) Hydrogen spectrum and (**E**) carbon spectrum was recorded on a Bruker Avance III 600 MHz NMR spectrometer (Bruker, Karlsruhe, Germany) with deuterated chloroform as solvent. (**F**) Molecular structure diagram of identified compound dolabella-3,7-dien-18-ol.

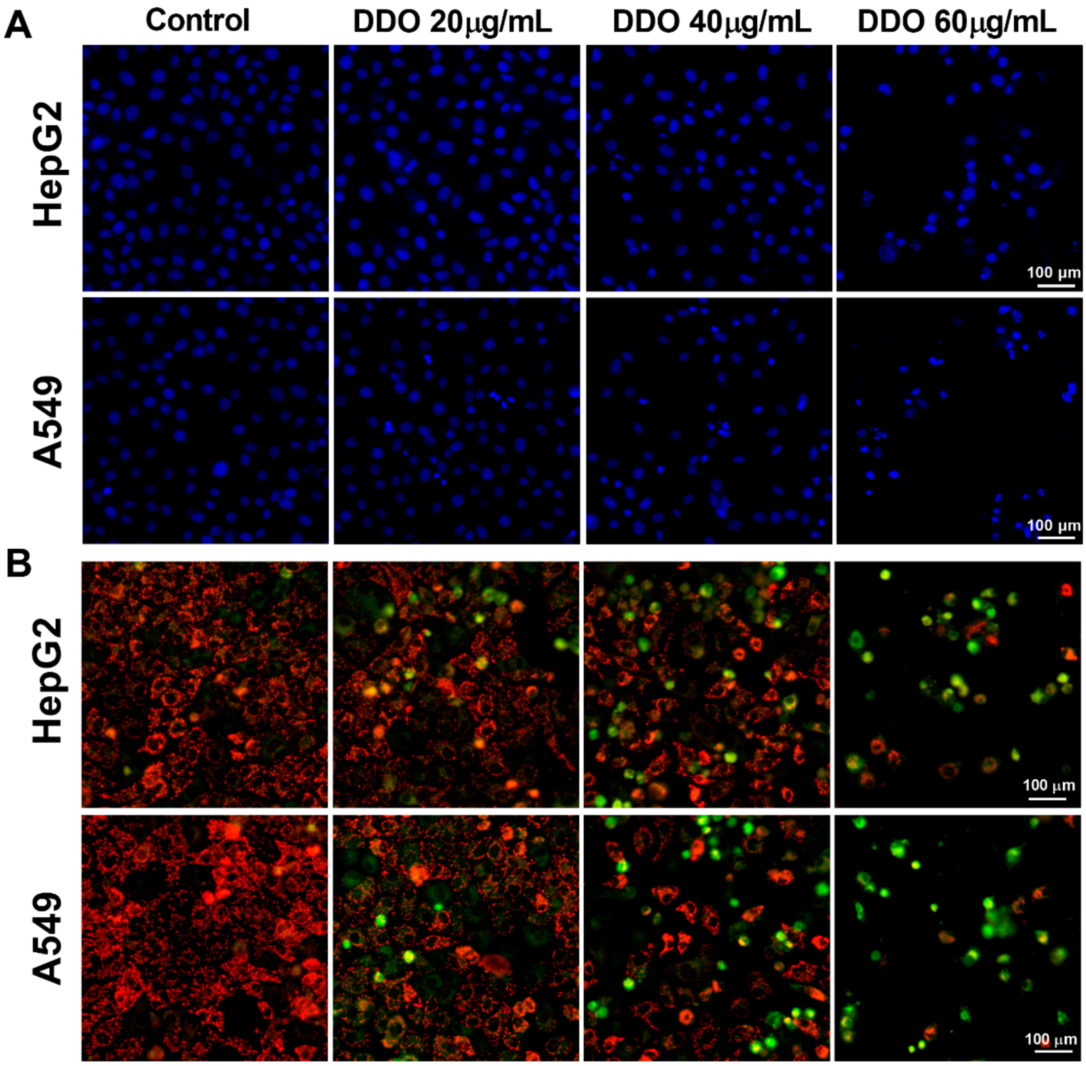


**Figure S2.** Effects of DDO on apoptosis and mitochondrial membrane potential of HepG2 and A549 Cells. HepG2 and A549 cells were treated with or without different concentrations of DDO for 24 h, then stained with (**A**) Hoechst 33342 or (**B**) JC-1. After washing twice with PBS, the cells were photographed using ImageXpress Micro Confocal High-Content Imaging System.

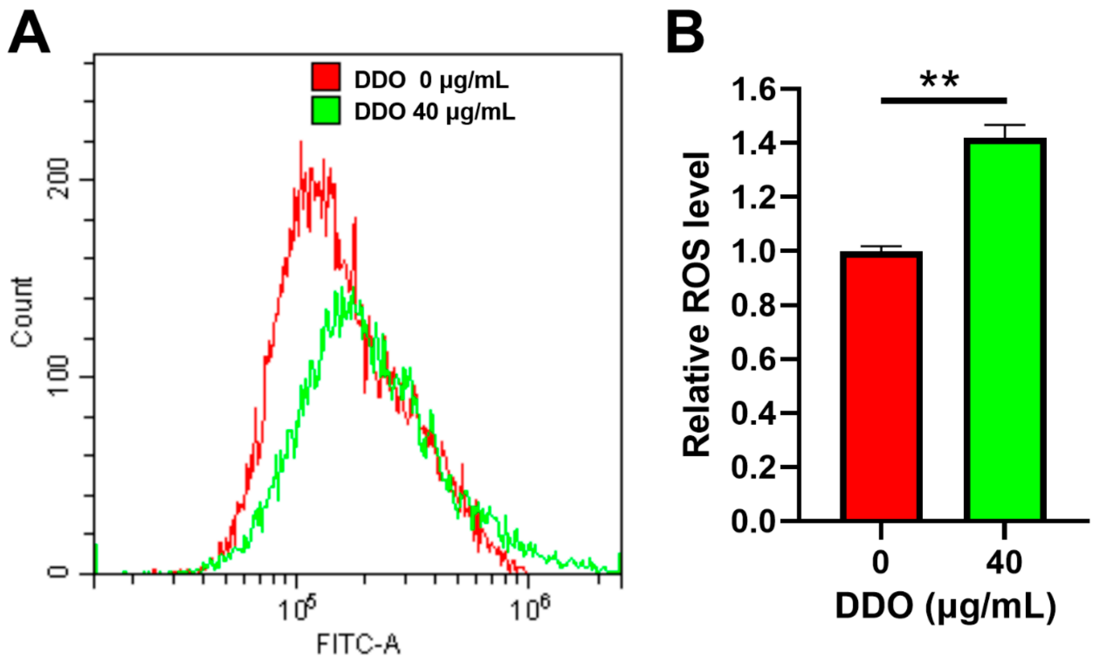


**Figure S3.** ROS levels of A549 cells treated by DDO. A549 cells were treated with DDO (40 μg/mL) for 24 h, then cells were collected and stained with DCFH-DA. (**A**) Fluorescence intensity was recorded on a Beckman Cytoflex S flow cytometer, and (**B**) relative fluorescence intensity was calculated; the values represent mean ± standard deviation of three independent experiments. ** $p$ <0.01.
